# Diagnostic Value of the lncRNA *NEAT1* in Peripheral Blood Mononuclear Cells of Patients with Sepsis

**DOI:** 10.1155/2017/7962836

**Published:** 2017-12-31

**Authors:** Shuying Huang, Kejian Qian, Yuanfang Zhu, Zikun Huang, Qing Luo, Cheng Qing

**Affiliations:** ^1^Department of Obstetrics and Gynecology, The First Affiliated Hospital of Nanchang University, Nanchang, Jiangxi 330006, China; ^2^Department of Nursing, Jiangxi Health Vocational College, Nanchang, Jiangxi 330052, China; ^3^Intensive Care Unit, The First Affiliated Hospital of Nanchang University, Nanchang, Jiangxi 330006, China; ^4^Department of Obstetrics and Gynecology, Shenzhen Baoan Maternal and Child Health Hospital, Shenzhen, Guangdong 518133, China; ^5^Department of Clinical Laboratory, The First Affiliated Hospital of Nanchang University, Nanchang, Jiangxi 330006, China

## Abstract

**Background:**

This study aims to evaluate the diagnostic value of nuclear-enriched abundant transcript 1 (*NEAT1*) expression in peripheral blood mononuclear cells (PBMCs) for the early diagnosis of sepsis.

**Methods:**

A total of 59 patients with sepsis, 52 noninfectious SIRS patients, and 56 healthy controls were recruited fort this study. The levels of *NEAT1* expression in PBMCs were measured using quantitative real-time polymerase chain reaction (qRT-PCR).

**Results:**

Compared with healthy controls, *NEAT1* expression of PBMCs in sepsis and SIRS groups were significantly increased (3.76 ± 0.71- and 1.64 ± 0.43-fold, resp.) (*P* < 0.01), but *NEAT1* levels are significantly lower in the SIRS group than in the sepsis group, and there was no statistical significant relevance between survivors and nonsurvivors in patients with sepsis. *NEAT1* with an area under the curve (AUC) of 0.851 (95% CI: 0.812–0.935) indicated sensitivity (67.85%) and specificity (87.27%) for the diagnosis for sepsis, the positive predictive value (PPV) was 83.3%, and the negative predictive value (NPV) was 71.6%. The AUC for *NEAT1* in the diagnosis of SIRS versus healthy controls was 0.755 (95% CI: 0.664–0.847), with 69.23% sensitivity and 70.91% specificity, the PPV was 72.3%, and the NPV was 72.49%.

**Conclusion:**

Measurement of *NEAT1* expression in PBMCs could be considered as a good additive marker for the diagnosis of sepsis.

## 1. Introduction

Sepsis is defined as a life-threatening organ dysfunction caused by a dysregulated host response to infection and is a common cause of death among patients in intensive care units. Sepsis can occur in patients with serious trauma, burns, multiple injuries, shock, or after major surgery, and it progresses rapidly from bacteremia to vital organ failure and even death. At present, sepsis is a major issue in the field of critical care medicine [1]. Currently, blood culture is the gold standard for the microbiological diagnosis of sepsis, but this method has drawbacks, including a lack of sensitivity owing to small sample volumes and the long time required for a confirmed diagnosis, resulting in delayed treatment and high mortality rates [2, 3]. To reduce the mortality rate for patients with sepsis, it is important to develop methods for early diagnosis, enabling prompt intervention.

Long noncoding RNAs (lncRNAs) are RNA polymerase II (RNAPII) transcripts that are longer than 200 nucleotides. They lack an open-reading frame, but utilize RNA–RNA, RNA–DNA, and RNA–protein interactions to regulate cell proliferation, apoptosis, damage, autophagy, and differentiation at transcriptional and posttranscriptional levels by splicing, degradation of nucleic acids, RNA capture, and translational interference [4]. They are of great significance in a number of diseases, including immune diseases [5], cancer [6], and cardiovascular diseases [7].

lncRNA nuclear-enriched abundant transcript 1 (*NEAT1*) was recently identified as an important regulator of cell functions; it interacts with many important regulators within cells and has roles in the formation, differentiation, and metastasis of neoplastic diseases, such as colon cancer [8], gall bladder cancer [9], ovarian cancer [10], and prostate cancer [11]. Notably, *NEAT1* is involved in the innate immunity response and is an important immunoregulatory factor. It is involved in the infection process in various infectious diseases, such as HIV in humans [12], hantavirus [13], and Zika virus [14]. Upregulated *NEAT1* expression has been observed in mononuclear cells of patients with systemic lupus erythematosus [15]. Furthermore, *NEAT1* can regulate the expression of IL-6 and CXCL10 inflammatory factors in THP-1 cells. We deduced that *NEAT1* may be involved in the immune response in sepsis based on the close relationships between IL-6 and CXCL10 cytokines and the inflammatory response in sepsis. However, previous studies have not examined the role of *NEAT1* in sepsis. Accordingly, in this study, *NEAT1* expression was examined in peripheral blood mononuclear cells (PBMCs) in patients with sepsis, noninfectious SIRS, and healthy volunteers to analyze its relationship with sepsis development and prognosis and to explore the diagnostic value and clinical significance of *NEAT1* in sepsis.

## 2. Materials and Methods

### 2.1. Patients and Healthy Controls

The diagnosis of sepsis and systemic inflammatory response syndrome (SIRS) was according to the American College of Chest Physicians and the Society of Critical Care Medicine (ACCP/SCCM) [16]. Exclusion criteria were as follows: individuals younger than 18 years old, patients suffering from immune diseases or receiving long-term glucocorticoids and immunosuppressive drugs, comorbid chronic liver and renal insufficiency, tuberculosis, AIDS, cancer, and patients who were pregnant. A total of 52 noninfectious SIRS patients (17 cases of compound injury, 8 cases of acute pancreatitis, 19 cases of systemic lupus erythematosus, 5 cases of thermoplegia, and 3 cases of organic phosphorus poisoning) and 59 sepsis patients at The First Affiliated Hospital of Nanchang University were selected for this study between May 2016 and December 2016. APACHE II scores, SOFA scores, absolute neutrophil counts, and procalcitonin (PCT) were evaluated in patients using blood samples collected at various time points. The survival statuses of patients were traced and recorded 28 days after their admission to the intensive care unit. A group of 56 healthy subjects was selected over the same period and served as normal controls. This study was approved by the ethics committee of the hospital, and written informed consent was obtained from all patients or from their authorized representatives.

### 2.2. Detection of NEAT1 in PBMCs

#### 2.2.1. Collection and Processing of Samples

Venous blood (5 mL) was collected in sodium heparin tubes in the morning before eating, mixed well, and left until use. PBMCs were isolated using the conventional Ficoll gradient method. Total RNA was extracted using the TRIzol (Invitrogen, Carlsbad, CA, USA) test kit following the manufacturer's instructions. The quality and concentration of total RNA were determined using a spectrophotometer.

#### 2.2.2. RT-qPCR Analysis

PrimeScript RT Master Mix (Takara, Dalian, China) was used for RT-qPCR and cDNA reverse transcription in accordance with the product manual. SYBR Premix EX Taq™ II (Takara) was used for qPCR; the ABI 7500 system (Applied Biosystems, Waters, MA, USA) was used for sample loading. The detection procedures and reaction criteria were set and carried out with reference to the instructions provided with the test kit. Primers were as follows: *NEAT1* forward, 5′-CTTCCTCCCTTTAACTTATCCATTCAC-3′; *NEAT1* reverse, 5′-CTCTTCCTCCACCATTACCAACAATAC-3′ [12]. *GAPDH* was used as an internal reference with the following primers: *GAPDH* forward, 5′-GCACCGTCAAGGCTGAGAAC-3′; *GADPH* reverse, 5′-TGGTGAAGACGCCAGTGGA-3′ [12]. Tests on all samples were run in triplicate. The relative expression of RNA was computed based on the 2^−ΔΔCt^ method.

#### 2.2.3. Enzyme-Linked Immunosorbent Assay

Serum C-reactive protein (CRP), PCT, and interleukin-6 (IL-6) were determined by enzyme-linked immunosorbent assays (BioSource, Kansas City, MO, USA) in accordance with the product manual.

### 2.3. Statistical Analysis

Statistical analyses were performed using GraphPad Prism (version 5). Quantitative results are expressed as means ± standard deviation. Differences in more than 2 group data were evaluated by the one-way ANOVA or Kruskal-Wallis analysis of variance by ranks. The *t*-test was used to compare the means of two populations with normally distributed values. The Mann–Whitney *U* test was used for comparisons between two groups for which data were not normally distributed. Qualitative data were tested using *χ*^2^ tests. The diagnostic value of *NEAT1* was analyzed by plotting a receiver operating characteristic (ROC) curve. *P* < 0.05 was considered statistically significant.

## 3. Results

### 3.1. General Clinical Information

In total, 52 noninfectious SIRS patients, 59 patients with sepsis, and 56 healthy volunteers were included in this study. As shown in Table 1, there were no differences in age and gender between these three groups. The levels of leukocytes, blood lactate, and CRP were significantly higher in patients with sepsis than in the SIRS and control groups. Also, the sepsis group had higher levels of PCT, SOFA score, APACHEII score, and mortality than the SIRS group (Table 1).

### 3.2. Analysis of Sepsis Survivors and Nonsurvivors

Further comparisons indicated that the SOFA scores, APACHE II scores, PCT, Lac, and CRP were significantly higher in sepsis nonsurvivors than in survivors (Table 2). However, there were no differences in leukocyte count and age between these two groups.

### 3.3. Expression of NEAT1 in PBMCs

**Figure 1:**
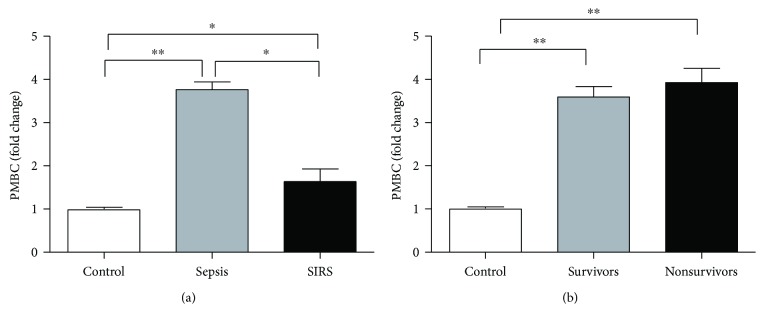
*NEAT1* expression is increased in patients with sepsis and SIRS. (a) Expression of *NEAT1* in PBMCs of patients with SIRS (*n* = 52), sepsis (*n* = 59), and normal controls (*n* = 56). (b) *NEAT1* expression in PBMCs of patients with sepsis who survived (*n* = 35) or who died (*n* = 24). ^∗^*P* < 0.05 and ^∗∗^*P* < 0.01.

qRT-PCR was used to detect differences in *NEAT1* expression in PBMCs among patients with SIRS, sepsis, and healthy volunteers. *NEAT1* levels in PBMCs were significantly higher (3.76 ± 0.71-fold) in patients with sepsis than in the control group, although SIRS group *NEAT1* levels are higher (1.64 ± 0.43-fold) than those of the control group, but significantly lower than those of the sepsis group (*P* < 0.01; Figure 1(a)). Upon further analysis, *NEAT1* expression levels in sepsis survivors and nonsurvivors were higher than those in the control group, but the difference between survivors and nonsurvivors was not significant (3.61 ± 0.62 versus 3.83 ± 0.57, *P* > 0.05; Figure 1(b)).

### 3.4. Diagnostic Value of NEAT1 for Sepsis

**Figure 2:**
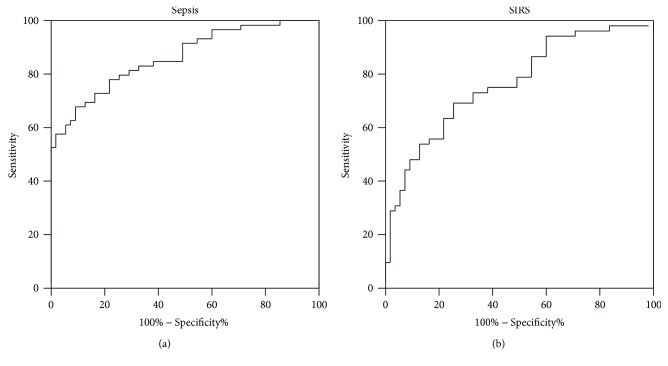
ROC curve analysis of NEAT1 concentrations for the prediction of sepsis and SIRS.

To determine the predictive value of *NEAT1*, an ROC curve analysis was performed. The area under the curve (AUC) for *NEAT1* in the diagnosis of sepsis versus healthy controls was 0.851 (95% CI: 0.811–0.920), and at a cut-off point set at 1.95 (Figure 2(a)), the sensitivity was 67.85% and specificity was 87.27%, and the positive predictive value (PPV) was 83.3% and the negative predictive value (NPV) was 71.6%. The AUC for *NEAT1* in the diagnosis of SIRS versus healthy controls was 0.755 (95% CI: 0.664–0.847), and at a cut-off point set at 1.45 (Figure 2(b)), with 69.23% sensitivity and 70.91% specificity, the PPV was 72.3% and the NPV was 72.49%.

## 4. Discussion

The pathogenesis of sepsis is complex and is affected by many factors. In addition to bacterial infection, viruses, fungi, parasites, and other pathogenic microorganisms can also cause sepsis. Hence, the clinical manifestations of sepsis are often nonspecific and can interfere with the clinical diagnosis [17]. PCT has clinical significance for diagnosing sepsis, evaluating treatment options, and assessing prognosis and is used extensively in clinical practice. However, PCT concentrations also increase in some noninfectious diseases, thus limiting its clinical applications to a certain degree [18]. Inflammatory markers, such as CRP, CD64, IL-6, and leukocytes, also have some diagnostic value. However, it is still not possible to identify and diagnose sepsis early owing to sensitivity and specificity issues [19]. Clinically, it is difficult to obtain a rapid and accurate diagnosis of sepsis using existing inflammatory markers or diagnostic methods. Moreover, a delay in the clinical diagnosis and treatment of sepsis can lead to the rapid development of circulatory failure, multiple organ dysfunction syndrome, and even death. There is a pressing need to discover new, effective biomarkers for the identification and diagnosis of sepsis to enable early intervention.

Tissue specificity is typically higher for lncRNAs expression than protein-coding mRNA expression, giving lncRNAs a potential advantage as diagnostic biomarkers. Many experimental studies have shown that lncRNAs can be potential biomarkers for the diagnosis of human diseases. In oncology, Wang and colleagues [20] found that the lncRNA MALAT1 is a biomarker for bladder carcinoma, Xie and his colleagues found that the lncRNA HULC is a biomarker for hepatocellular carcinoma [21], and Zhou and colleagues [22] found that the lncRNA H19 is a possible diagnostic marker for gastric cancer [22]. With respect to cardiovascular diseases, Zhang and colleagues [23] found that the lncRNA MHRT in the plasma could potentially be an early diagnostic biomarker for acute myocardial infarction and Kumarswamy et al. [24] found that the lncRNA LIPCAR can be a diagnostic marker for patients with heart failure. However, there are no studies of lncRNA biomarkers for the diagnosis of sepsis to date.

Based on an extensive literature review, the lncRNA molecule *NEAT1* was identified as a candidate biomarker for sepsis. Previous studies have shown that *NEAT1* is expressed in the nucleus. *NEAT1* can combine with p54nrb, PSF, and PSPpl (three RNA-binding proteins) to form paraspeckles. And *NEAT1* is required for these interactions as the backbone for the paraspeckle structure [25, 26]. It has two subtypes, *NEAT1–1* (3.7 kb) and NEAT1–2 (23 kb). Studies have proven that *NEAT1–1* is the main subtype expressed in PBMCs [15], HeLa cells [27], and various organs in mice [28]. Therefore, for the purpose of this study, we directly measured *NEAT1–1* expression in the PBMCs of patients with sepsis.

In this study, we found higher NEAT1 expression in the PBMCs of patients with sepsis than in healthy volunteers by qRT-PCR, although *NEAT1* levels in the SIRS group are higher than those in the control group, but significantly lower than the set of sepsis, indicating that increased inflammation can lead to higher levels of *NEAT1*. According to previous studies, human umbilical vein endothelial cells (HUVECs) infected with hantavirus exhibit increased *NEAT1–1* expression. Transfection with upregulated *NEAT1–1* promoted the secretion of the inflammatory factor IFN in these cells; this mechanism is associated with the activation of the RIG-I/IRF7 signaling pathway [14]. In a study of immune function of patients with SLE [15], *NEAT1* expression was increased in the PBMCs of these patients; *NEAT1* expression increases rapidly in PBMCs by stimulation with LPS in vitro, and peak levels are reached at 2 h, compared with 12–48 h required for PCT to reach peak levels [29]. These results indicated that *NEAT1–1* is an early inflammatory response factor. Furthermore, the study indicated that *NEAT1* promotes inflammatory responses in PBMCs by activating the JNK/ERK MAPK signaling pathway. These studies suggest that *NEAT1* is a proinflammatory molecule. In our study, *NEAT1* was significantly upregulated in the PBMCs of patients with sepsis, consistent with the excessive inflammatory response during sepsis observed in clinical studies. In addition, *NEAT1* can be upregulated more rapidly at the early stages of inflammation than PCT. Therefore, *NEAT1* is a potentially effective marker for rapid sepsis diagnosis.

There was no difference in NEAT1 expression between sepsis survivors and nonsurvivors, showing that it has no predictive value for mortality of sepsis. Further ROC curve analysis revealed that NEAT1 has clinical value for sepsis diagnosis, which is more useful for SIRS diagnosis. Considering that the cut-off point is set at 1.95 and 1.45 in the sepsis and SIRS groups, it is possible to be identified as indicators of the two diseases in the future but still needs a lot of research to confirm. Moreover, the PCT, SOFA scores, and APACHE II scores were higher in the sepsis group than in the healthy individuals, and these differences could be explained by the inclusion of patients who were relatively older, with more serious conditions in the sepsis group.

Unfortunately, owing to the small clinical sample size, we were unable to evaluate the use of NEAT1 in the early diagnosis of sepsis nor were we able to examine different stages of sepsis in patients of various ages, genders, comorbidities, or pregnancy status; hence, it was not possible to compare NEAT1 with PCT or other traditional molecular markers of sepsis. We also did not conduct an in-depth study of the molecular mechanisms linking NEAT1 to sepsis. These limitations will guide future in-depth studies of the use of NEAT1 as a molecular marker for sepsis diagnosis.

In conclusion, upregulated NEAT1 expression in patients with sepsis was discovered for the first time in our study, indicating an association between NEAT1 and immune dysfunction in patients with sepsis. NEAT1 is a potential molecular marker for sepsis diagnosis, offering a new option for early diagnosis. Of course, extensive research is still needed to enable the practical applications of NEAT1.

## Figures and Tables

**Table 1:** Baseline characteristics of the study population.

	Sepsis	SIRS	Healthy controls	*P* value
Number	59	52	56	—
Age (years)	58.7 ± 14.6	54.9 ± 15.9	52.6 ± 17.7	0.19
Sex (male/female)	35/24	30/22	33/23	0.73
WBC (×10^9^/L)	13.57 ± 6.63^∗^	8.21 ± 3.85	6.57 ± 1.36	<0.01
PCT (ng/mL)	8.9 ± 1.87	0.6 ± 0.12	—	<0.01
SOFA score	8.7 ± 3.2	4.6 ± 2.3	—	<0.01
APACHEII score	17.8 ± 5.7	8.7 ± 3.5	—	<0.01
Lac (mmol/L)	3.98 ± 2.13^∗^	0.65 ± 0.01	0.51 ± 0.00	<0.01
CRP (*μ*g/L)	17.59 ± 4.87^∗^	2.75 ± 1.24	2.08 ± 1.01	<0.01
Survival at 28 d, *n* (%)	35 (59.3%)^∗^	47 (90.3%)	0	<0.01

^∗^ *P* < 0.05, versus SIRS.

**Table 2:** Clinical data for survivors and nonsurvivors based on a 28-day survival.

	Survivors (*n* = 23)	Nonsurvivors (*n* = 12)	*P* value
Age (years)	53.6 ± 17.1	50.1 ± 15.3	0.242
SOFA score	6.1 ± 2.3	10.4 ± 3.5	<0.001
APACHE II score	13.1 ± 5.5	19.7 ± 7.8	<0.001
WBC (×10^9^/L)	13.57 ± 6.63	16.57 ± 1.36	0.158
PCT (ng/mL)	3.2 ± 2.64	7.8 ± 2.93	<0.001
Lac (mmol/L)	2.58 ± 1.01	4.01 ± 1.90	<0.001
CRP (*μ*g/L)	12.33 ± 4.67	20.68 ± 5.94	<0.001

## References

[1] Graetz T. J., Hotchkiss R. S. (2017). Sepsis: preventing organ failure in sepsis – the search continues. *Nature Reviews Nephrology*.

[2] Ho J., Chan H., Wong S. H. (2016). The involvement of regulatory non-coding RNAs in sepsis: a systematic review. *Critical Care*.

[3] Correia C. N., Nalpas N. C., McLoughlin K. E. (2017). Circulating microRNAs as potential biomarkers of infectious disease. *Frontiers in Immunology*.

[4] Beermann J., Piccoli M. T., Viereck J., Thum T. (2016). Non-coding RNAs in development and disease: background, mechanisms, and therapeutic approaches. *Physiological Reviews*.

[5] Satpathy A. T., Chang H. Y. (2015). Long noncoding RNA in hematopoiesis and immunity. *Immunity*.

[6] Jiang C., Li X., Zhao H., Liu H. (2016). Long non-coding RNAs: potential new biomarkers for predicting tumor invasion and metastasis. *Molecular Cancer*.

[7] Peters T., Hermans-Beijnsberger S., Beqqali A. (2016). Long non-coding RNA Malat-1 is dispensable during pressure overload-induced cardiac remodeling and failure in mice. *PLoS One*.

[8] Li Y., Li Y., Chen W. (2015). NEAT expression is associated with tumor recurrence and unfavorable prognosis in colorectal cancer. *Oncotarget*.

[9] Parasramka M., Yan I. K., Wang X. (2017). BAP1 dependent expression of long non-coding RNA NEAT-1 contributes to sensitivity to gemcitabine in cholangiocarcinoma. *Molecular Cancer*.

[10] Chen Z. J., Zhang Z., Xie B. B., Zhang H. Y. (2016). Clinical significance of up-regulated lncRNA NEAT1 in prognosis of ovarian cancer. *European Review for Medical and Pharmacological Sciences*.

[11] Chakravarty D., Sboner A., Nair S. S. (2014). The oestrogen receptor alpha-regulated lncRNA NEAT1 is a critical modulator of prostate cancer. *Nature Communications*.

[12] Jin C., Peng X., Xie T. (2016). Detection of the long noncoding RNAs nuclear-enriched autosomal transcript 1 (NEAT1) and metastasis associated lung adenocarcinoma transcript 1 in the peripheral blood of HIV-1-infected patients. *HIV Medicine*.

[13] Ramaiah A., Contreras D., Gangalapudi V., Padhye M. S., Tang J., Arumugaswami V. (2016). Dysregulation of long non-coding RNA (lncRNA) genes and predicted lncRNA-protein interactions during Zika virus infection. *bioRxiv*.

[14] Ma H., Han P., Ye W. (2017). The long noncoding RNA NEAT1 exerts antihantaviral effects by acting as positive feedback for RIG-I signaling. *Journal of Virology*.

[15] Zhang F., Wu L., Qian J. (2016). Identification of the long noncoding RNA NEAT1 as a novel inflammatory regulator acting through MAPK pathway in human lupus. *Journal of Autoimmunity*.

[16] Levy M. M., Fink M. P., Marshall J. C. (2003). 2001 SCCM/ESICM/ACCP/ATS/SIS international sepsis definitions conference. *Critical Care Medicine*.

[17] Lever A., Mackenzie I. (2007). Sepsis: definition, epidemiology, and diagnosis. *BMJ*.

[18] Dou Y. H., Du J. K., Liu H. L., Shong X. D. (2013). The role of procalcitonin in the identification of invasive fungal infection—a systemic review and meta-analysis. *Diagnostic Microbiology and Infectious Disease*.

[19] Jekarl D. W., Lee S. Y., Lee J. (2013). Procalcitonin as a diagnostic marker and IL-6 as a prognostic marker for sepsis. *Diagnostic Microbiology and Infectious Disease*.

[20] Wang X. S., Zhang Z., Wang H. C. (2006). Rapid identification of UCA1 as a very sensitive and specific unique marker for human bladder carcinoma. *Clinical Cancer Research*.

[21] Xie H., Ma H., Zhou D. (2013). Plasma HULC as a promising novel biomarker for the detection of hepatocellular carcinoma. *BioMed Research International*.

[22] Zhou X., Yin C., Dang Y., Ye F., Zhang G. (2015). Identification of the long non-coding RNA H19 in plasma as a novel biomarker for diagnosis of gastric cancer. *Scientific Reports*.

[23] Zhang J., Gao C., Meng M., Tang H. (2016). Long noncoding RNA MHRT protects cardiomyocytes against H_2_O_2_-induced apoptosis. *Biomolecules & Therapeutics*.

[24] Kumarswamy R., Bauters C., Volkmann I. (2014). Circulating long noncoding RNA, LIPCAR, predicts survival in patients with heart failure. *Circulation Research*.

[25] Bond C. S., Fox A. H. (2009). Paraspeckles: nuclear bodies built on long noncoding RNA. *The Journal of Cell Biology*.

[26] Clemson C. M., Hutchinson J. N., Sara S. A. (2009). An architectural role for a nuclear noncoding RNA: *NEAT1* RNA is essential for the structure of paraspeckles. *Molecular Cell*.

[27] Sasaki Y. T., Ideue T., Sano M. (2009). MEN*ε*/*β* noncoding RNAs are essential for structural integrity of nuclear paraspeckles. *Proceedings of the National Academy of Sciences of the United States of America*.

[28] Nakagawa S., Naganuma T., Shioi G., Hirose T. (2011). Paraspeckles are subpopulation-specific nuclear bodies that are not essential in mice. *The Journal of Cell Biology*.

[29] Dandona P., Nix D., Wilson M. F. (1994). Procalcitonin increase after endotoxin injection in normal subjects. *The Journal of Clinical Endocrinology and Metabolism*.

